# Laryngeal Electromyography and Acoustic Voice Analysis in Parkinson's Disease: a comparative study

**DOI:** 10.1590/S1808-86942010000100008

**Published:** 2015-10-17

**Authors:** Ana Paula Zarzur, Isabella Sebusiani Duarte, Gabrielle do Nascimento Holanda Gonçalves, Maria Angela Ueda Russo Martins

**Affiliations:** 1 Master's degree in otorhinolaryngology, medical assistant; 2 Trained in otorhinolaryngology, otorhinolaryngologist; 3 Undergraduate medical course, medical resident of otorhinolaryngology; 4 Speech therapist, head of the Speech Therapy Unit, Beneficencia Portuguesa Hospital, Sao Paulo

**Keywords:** acoustics, voice disorders, parkinson disease, electromyography, larynx

## Abstract

Parkinson's disease (PD) involves a progressive depletion of dopamine in the basal ganglia leading to motor alterations. Oral communication impairment occurs in 75% to 90% of patients and has been poorly studied.

**Aim:**

to asses laryngeal electromyography (LEMG) patterns and correlate them to vocal analysis in patients with Parkinson's disease.

**Materials and Methods:**

This is a prospective study. Twenty six adults with PD underwent laryngeal electromyography. Rest and phonation potentials were analyzed. VOXMETRIA® and GRAM 5.1.6. ® were used in acoustic analysis.

**Results:**

The main electromyographic pattern observed in the PD group was rest hypertonicity meaning that patients with PD presented with spontaneous intrinsic laryngeal muscle activity during voice rest, which occurred in 73% of the individuals. Not a case of laryngeal tremor was detected by electromyography, although vocal tremor was detected by VOXMETRIA in 69.5% of the individuals and in 61% of them by perceptive-auditive analysis.

**Conclusion:**

Vocal tremor was the main acoustic change in the PD group, with no correlation to LEMG findings.

## INTRODUCTION

Parkinson's disease is caused by dopaminergic deficiencies in the basal nuclei that generate motor actions.

According to Robbins, 70 to 92% of these patients progress with diseases of the tongue, larynx and pharynx. Oral communication is the main complaint in 30% of patients.^1^

Parkinson's dysarthria is characterized by abnormal voice modulation, hoarseness, voice tremor, decrease loudness and monotone voice. This diseases requires patients to constrict their vocal tract to a greater degree in order to issue certain phonemes.^1^

Electromyography is widely described in the literature as the diagnostic and monitoring method for systemic neuromuscular involvement in Parkinson patients. It was included as a diagnostic and therapeutic tool for laryngeal and voice disorders in the late 1980s.^2^,^3^

There are few references about the use of this method for studying laryngeal muscles in patients with Parkinson's disease. These studies have assumed that voice dysfunction was caused by the motor control peculiarities of this disease.

The purpose of this study was to define contractile reference standards for the intrinsic muscles of the larynx and to correlate them to the voice patters of Parkinson's disease patients with verbal communication complaints.

## SERIES AND METHOD

This study was presented to the institutional review board which approved it on July 2003.

The study group (Parkinson-PK) consisted of 26 subjects with Parkinson's disease, comprising 18 males and 8 females aged from 58 to 81 years (mean −68.8 years) from a neurology clinic. All had a final diagnosis for at least one month and used several drugs.

**Figure 1:**
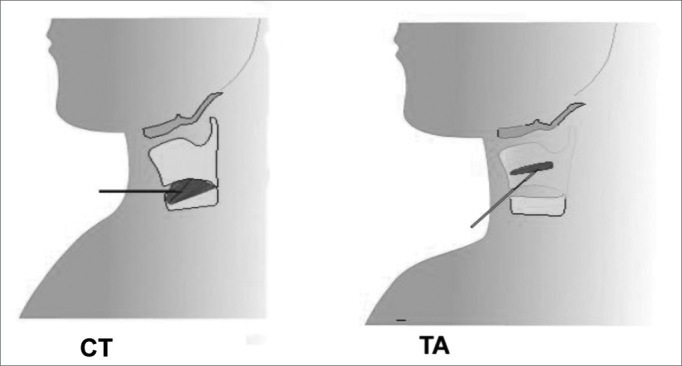
Electrode insertion in CT (cricothyroid) and TA (thyroarytenoid) muscles

Laryngeal electromyography (LEMG) was done at the electrophysiology unit. The thyroarytenoid (TA) and cricothyroid (CT) muscles were selected for this study because of their predominant action on voice and easy access (Fig. 1).

Action potentials during rest and phonation were measured; phonation tasks were the same throughout: issuing the vowel /i/ in treble and bass tones, during ten seconds each, between which a half minute rest of voice was given. One side only was investigated in each subject, and was chosen randomly.

A Medelec Teca model TD-50 monochannel electromyography unit with Rochester monopole electrodes with a 37 mm length and 2.7 mm gauge needle was used. Surface ground and reference electrodes were placed on the clavicular area one to two centimeters from the area of study. The monochannel electromyography unit and the monopolar electrode made it possible to study several action potentials simultaneously, one muscle at a time.

The electrical potentials were measured with patient in dorsal decubitus, relaxed, with the neck slightly hype-rextended, with no topical anesthesia, after local asepsis with 70% alcohol. Anatomical parameters guided needled electrode insertion, as described in the literature, and the experience of the examiner with anatomical specimen dissections.^3^, ^4^, ^5^, ^6^

The needled electrode was first allocated to the CT muscle, perpendicular to the cricoid cartilage, 1.5cm from the midline.

The position of the electrode was checked in recordings of the electrical signal on the electromyography unit by asking subjects to issue the sound /i/ acute for 10 seconds. Next, voice rest was given for 30 seconds, followed by the bass tone for 10 seconds. Some patients were unable to sustain voice emission for 10 seconds; in these cases, maximum issuing time was measured.

The electrode was then redirected 30 degrees laterally and 45 degrees superiorly without being removed, transfixing the cricothyroid membrane to investigate the TA muscle. The position of the electrode was checked as described above for the CT muscle.

There were two results for each subject, one for the CT and another for the TA muscle.

Normal rest tracings were those with no electrical activity or with one or two motor unit potentials at a triggering rate of two to five per second.^7^

Muscle contraction tracings were those lasting from 5 to 6 ms and amplitudes between 200 and 500 uV, as reported in the literature for laryngeal muscles with monopolar electrodes.^7^

Hypercontractility tracings were those over muscle contraction values during phonation, or normal contraction values at rest.^8^

Parkinsonian tremor was considered as an oscillatory 3 to 7 Hz rhythmic contraction during muscle rest in different muscles.^9^

Tremor was investigated using acoustic analysis and auditory-perceptive assessment by two experienced speech therapists.

Acoustic voice analysis was done using the softwares VOXMETRIA® and GRAM 5.1.6®. Patients were seated with a microphone at a fixed distance of 15 cm at 45 degrees from the horizontal plane in a room with controlled environmental noise. Each patient issued the sustained vowel /a/ during maximum phonation time.

Electromyography and voice study results were analyzed comparatively.

## RESULTS

Voice tremor was detected in the spectrogram tracings (VOMETRIA and GRAM) in 69.5% of subjects; it was also present in the perceptive-auditory assessment in 61% of subjects.

**Graph 1:**
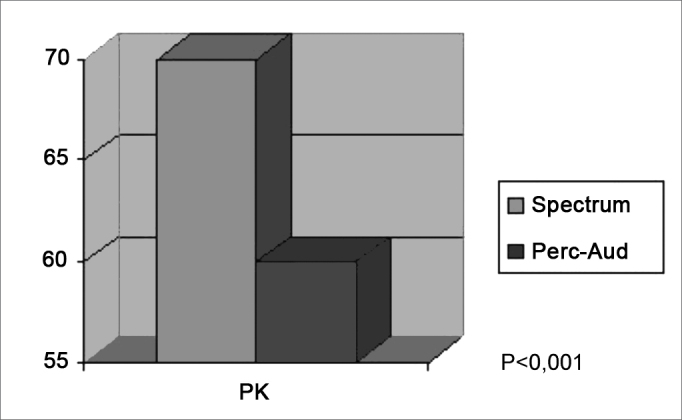
Voice analysis (spectrogram and perceptive-auditory) in the Parkinson group.

The spectrogram and the perceptive-auditory analysis identified voice tremor with a p<0,001 statistical significance (Graph 1), compared to LEMG.

**Figure 2:**
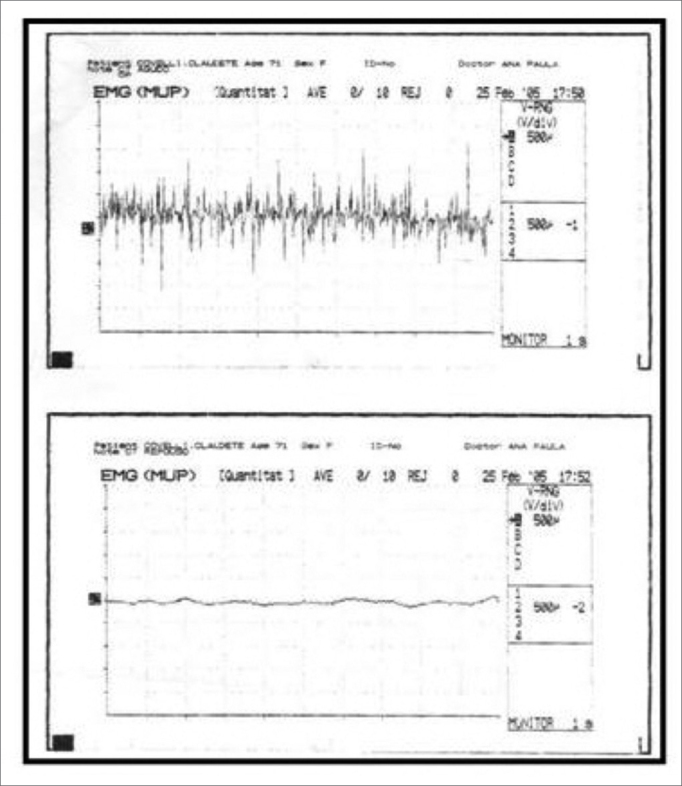
Normal laryngeal electromyography tracing during phonation (above) and rest (below).

**Figure 3:**
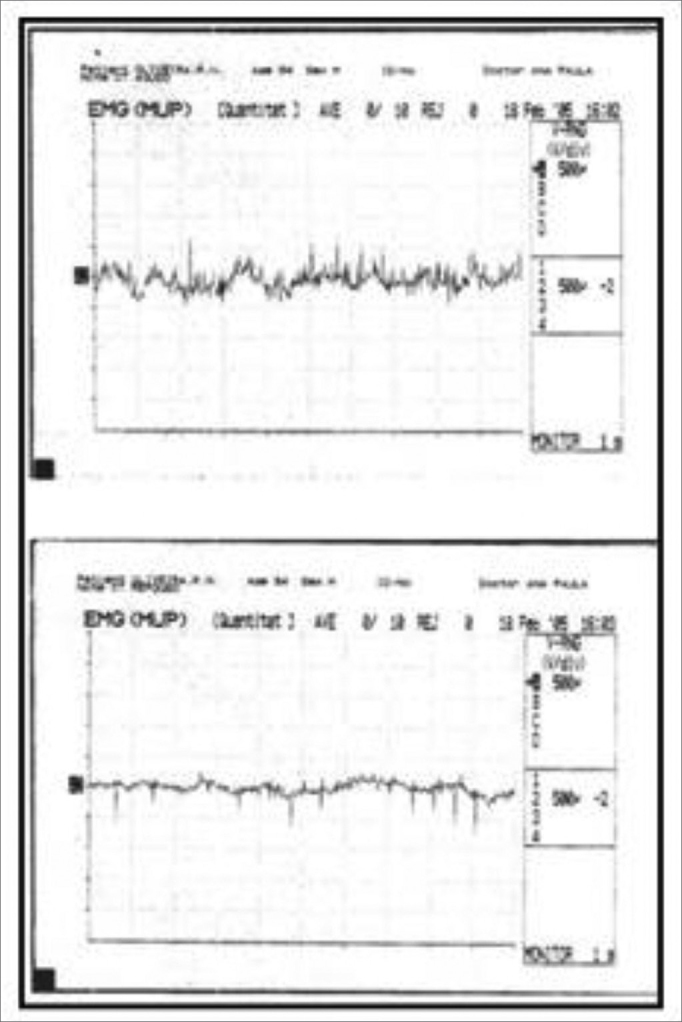
Altered laryngeal electromyography during voice rest (lower tracing): several action potentials (muscle activity) are seen.

LEMG revealed hypercontractility during voice rest in 73% of subjects in the study group. No tremor cases were detected (Figs. 2 and 3). Findings were statistically similar for CT and TA muscles in all subjects.

The statistical analysis (chi-square and Fisher's test) showed no correlation between LEMG and voice analysis for detecting tremor in patients.

## DISCUSSION

Dysphonia affects the social and professional lives of individuals. Quality of life impact studies show significant limitations that justify treating these symptoms.^10^

Among the limitations of patients with Parkinson's disease, verbal communication difficulties are among the most important and prevalent.^1,^^11,^^12^

Electromyography is currently used in many studies of Parkinson's disease in muscle groups other than laryngeal muscles.^13^,^14^

Percutaneous LEMG provides easy access to the CT and TA muscles.^4^

In 19 of 26 patients with Parkinson's disease (73%) involuntary motor activity during voice rest was observed, which may be explained as increased susceptibility of alpha-motor neurons to stimuli from supra-segmental structures in Parkinsonian patients; this translates into a lack of complete relaxation, or muscle hypertonia, in these patients. Muscle rigidity in patients with Parkinson's disease may reduce the respiratory volume; poor coordination increases transglottic air leakage, altering pneumo-phonoarticulatory coordination.^18^ Hypercontractility at rest, seen in LEMG, would correspond to Parkinsonian rigidity.

Zarzur et al. (2007) showed that hypercontractility at rest appears to be a feature of the group with disease.^8^ This finding may be supportive in the differential diagnosis with other laryngeal dysmotility conditions, such as spastic dysphonia and essential tremor. The features of electromyography tracings found in other neurological dysmotility conditions are different.

LEMG did not reveal laryngeal tremor in our study; this was evidenced in the acoustic and perceptive-auditory analysis (69.5% and 61%). We conclude that dysarthro-phonia in Parkinsonian patients shows changes of the respiratory, resonance and articulatory systems, and not only voice.^17^ Treatment, therefore, should include dopaminergic drugs and specific physical and speech therapy, the results of which may be monitored.

## CONCLUSION

This study shows up to this point that the predominant laryngeal electromyography pattern in Parkinsonian patients is hypercontractility at rest, with tremor evidenced only during voice analysis, without electromyography findings.
